# Supplementary material for: ZBED1 Regulates Genes Important for Multiple Biological Processes of the Placenta
Source: Genes (Basel). 2022 Jan 12;13(1):133. doi: 10.3390/genes13010133 (PMC8775481; doi:10.3390/genes13010133)
Supplement: Supplementary file 1 [file genes-13-00133-s001.zip › Figure S1.pdf]

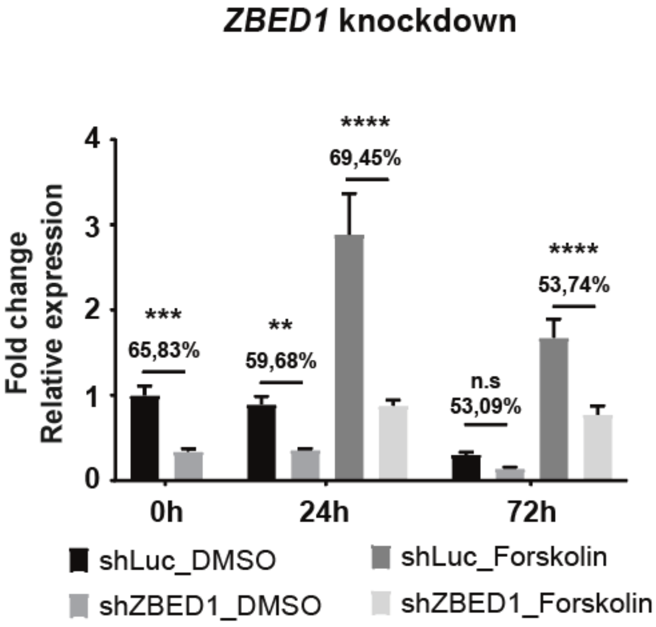

Figure S1. Validation of *ZBED1* knockdown in BeWo cells. BeWo cells were transduced with *ZBED1*-specific shRNA (shZBED1) or luciferase-specific shRNA (shLuc; negative control) and treated with forskolin or DMSO (control). Knockdown of *ZBED1* was validate by qPCR. The data is presented as mean ± SEM. Two-way ANOVA with following multiple comparison test of the mean values of the individual timepoints were used to calculate significance (n=3). ns = non-significant. **** P < 0.00001; *** P < 0.0001; ** P < 0.001.
