# Supplementary material for: ZBED1 Regulates Genes Important for Multiple Biological Processes of the Placenta
Source: Genes (Basel). 2022 Jan 12;13(1):133. doi: 10.3390/genes13010133 (PMC8775481; doi:10.3390/genes13010133)
Supplement: Supplementary file 1 [file genes-13-00133-s001.zip › Figure S2.pdf]

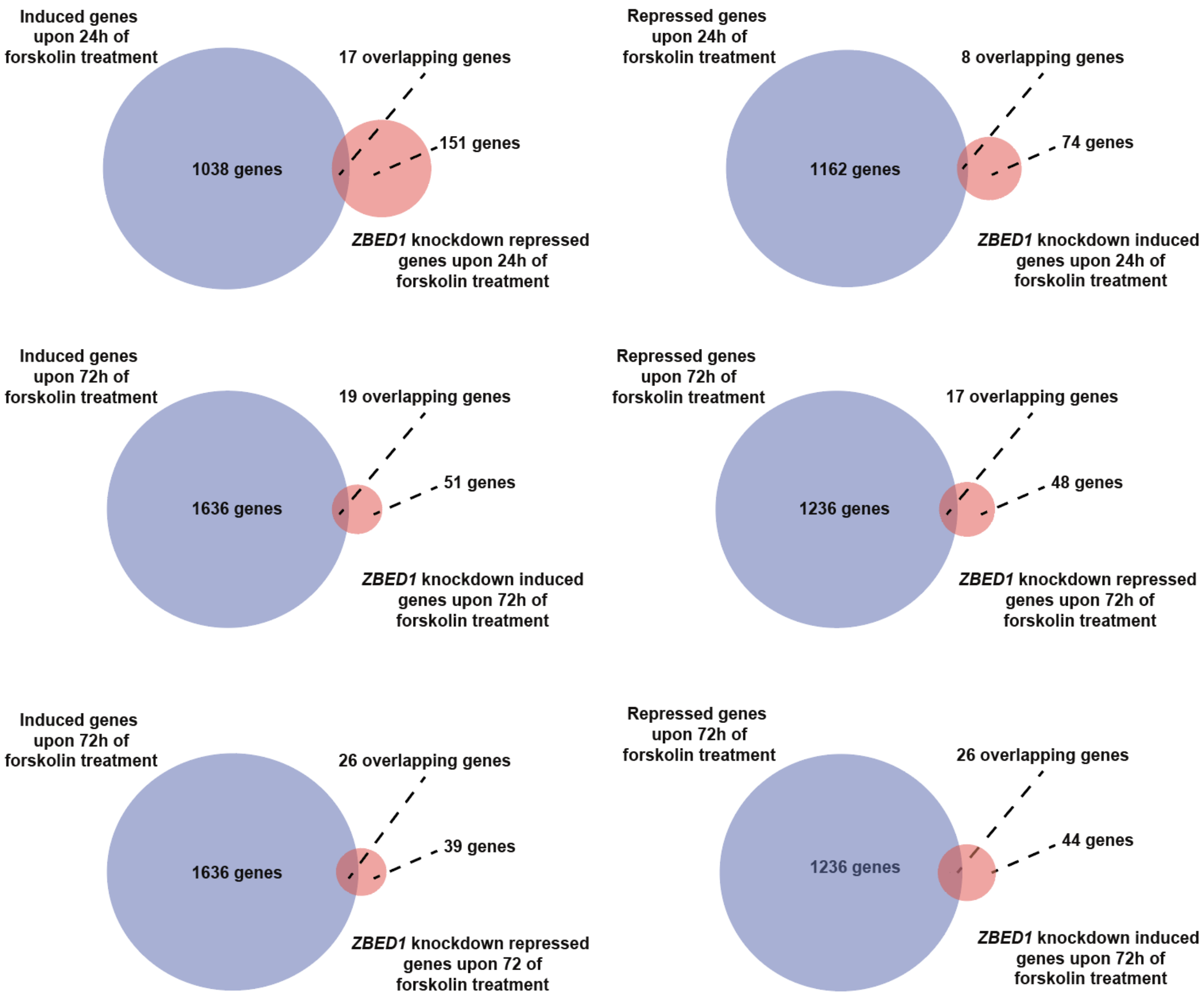

Figure S2. ZBED1 regulates crucial genes of biological processes related to BeWo differentiation. Overlap of genes regulated by forskolin treatment (purple) and ZBED1 knockdown during forskolin treatment (pink) (RNA-seq data).
